# Bulk Oriented UHMWPE/FMWCNT Films for Tribological Applications

**DOI:** 10.3390/polym9110629

**Published:** 2017-11-19

**Authors:** Aleksey V. Maksimkin, Saidkhuja G. Nematulloev, Dilyus I. Chukov, Vladimir D. Danilov, Fedor S. Senatov

**Affiliations:** 1 National University of Science and Technology “MISIS”, Moscow 119049, Russia; nematulloev_said@mail.ru (S.G.N.); dil_chukov@mail.ru (D.I.C.); senatov@misis.ru (F.S.S.); 2 Tambov State Technical University, Tambov 392000, Russia; 3 Mechanical Engineering Research Institute of the Russian Academy of Sciences, Moscow 101990, Russia; danilovvd@mail.ru

**Keywords:** UHMWPE, films, supramolecular structure, strength, coefficient of friction, wear rate

## Abstract

Bulk oriented films based on ultrahigh molecular weight polyethylene (UHMWPE) with a drawing ratio of 35 were prepared by using a low solvent concentration. Bulk oriented films were filled with fluorinated multi-walled carbon nanotubes (FMWCNTs). The structure of bulk oriented films on UHMWPE, which were manufactured at different stages of orientation, was investigated by scanning electron microscope (SEM) and differential scanning calorimetry (DSC). The addition of FMWCNTs at a concentration of 0.05 wt % in bulk oriented UHMWPE films led to an increase in the tensile strength by 10% (up to 1020 ± 23 MPa) compared to unfilled oriented films. However, the addition of FMWCNTs at a concentration of more than 0.5 wt % led to a decrease in tensile strength due to excessive accumulation of nanotubes and hindering of self-diffusion of UHMWPE macromolecules. The multiple increase in tensile strength, doubling the hardness, the formation of fibrillar structure, and the presence of carbon nanotubes led to a significant increase in tribological properties in bulk oriented films. Bulk oriented UHMWPE/1% FMWCNT films can be operated at a maximum contact pressure that is 18 times higher and exhibit a specific wear rate more than an order of magnitude and less than the traditional UHMWPE of isotropic structure. Bulk oriented UHMWPE/1% FMWCNT films have an extremely low dry coefficient of friction (COF) of 0.075 at a contact pressure of 31 MPa. The developed bulk oriented films can be used for manufacturing frictional surfaces for sliding bearings, or for acetabular cups for knee and hip endoprostheses.

## 1. Introduction

Ultrahigh molecular weight polyethylene (UHMWPE) is a unique material that is widely used in a variety of fields: medicine (acetabular cups for knee and hip endoprostheses [1], bone scaffolds [2], medical grade UHMWPE fiber, etc.), ballistic protections (protective garment, armor vests, helmets), sports, and industrial tribology. The most significant properties of UHMWPE are high biocompatibility, unique mechanical properties of highly oriented fibers and films, and antifriction, which includes the ability to work under dry sliding conditions, as it exhibits a low friction coefficient and a high wear resistance.

Polyethylene chains have a covalent C–C bond with a very high energy of 335 kJ/mol. Extended polyethylene chains are potentially very stiff and strong. Therefore, there is a special main interest in obtaining materials based on UHMWPE with an oriented supramolecular structure. The ultimate strength and elastic modulus of composites based on UHMWPE with isotropic structure do not exceed 50 MPa and several GPa, respectively. On the other hand, ultra-oriented fibers based on UHMWPE have a tensile strength of 3.3–3.9 GPa and an elastic modulus of 110–140 GPa [3,4]. The tribological properties of UHMWPE can also be greatly improved by its orientation of structure. Reference [5] shows that the dry friction coefficient was reduced from 0.20 to 0.15 and wear was reduced by 44% after the obtaining of nanofibrillar structure of UHMWPE. Reference [6] shows that the friction coefficient in lubricant (distillated water) decreased from 0.09 to 0.05, and wear decreased by a factor of ~3 after obtaining the oriented supramolecular structure in UHMWPE. According to DSM (Dyneema, Geleen, The Netherlands) highly oriented UHMWPE fibers have a recorded low dry and wet friction coefficient of 0.04 [7]. Therefore, there is a special main interest in obtaining materials based on UHMWPE with a high degree of orientation of macromolecules for the further manufacturing of various tribological products that can be used in technical engineering and implantology. For example, materials based on UHMWPE with a high degree of orientation can be used to form friction surfaces in products such as sliding bearings and acetabular cups for knee and hip endoprostheses. However, in order to produce such products, it is necessary to obtain bulk highly oriented films based on UHMWPE with a width of more than 2 mm. Currently, commercial UHMWPE fibers produced by gel-forming technology have a diameter of 9 to 20 μm (such as Dyneema Fiber). Therefore, it is difficult to produce a uniform friction surface by using fibers with such a small diameter.

Obtaining highly oriented materials based on UHMWPE is considered technologically difficult. UHMWPE has a high molecular mass (more than 10^6^ g/mol) and a high melt flow index in the molten state. The high concentration of entanglement coupling with macromolecules causes a high overstrain in the polymer chains during their orientation at a temperature close to the melting temperature of the polymer, which leads to a precocious rupture of macromolecules and the impossibility of achieving a high draw ratio. Therefore, UHMWPE fibers cannot be prepared by melt spinning.

Gel-spinning technology is widely used for the processing of UHMWPE into highly oriented fibers [8,9]. Fiber precursors are prepared by using a gel-spinning process. They have a lamellar structure and can be stretched 100 times or more by hot drawing. The main disadvantage of this method is the need to use large amounts of solvents such as: Vaseline oil, xylene, decalin, etc. Therefore, the cost of point-end product increases and the problems associated with solvent utilization arise.

There is another approach to obtaining highly oriented fibers: using solid-state processing. This method is based on the formation of an optimal supramolecular structure during the synthesis stage of UHMWPE reactor powder, which leads to a high drawability [10,11,12]. It permits one to decrease the solvent concentration or even to exclude the solvent usage. This approach has been used in this research.

One of the common ways to improve the tribological properties of polymeric materials is the use of antifriction disperse fillers. Carbon nanotubes are a promising antifriction filler which have an exceptional wear resistance [13]. They behave as a lubricant [14,15]. Nanotubes have a theoretical ultimate strength of tens of GPa and their elastic modulus is in TPa range. Their high aspect ratio plays a role in increasing the load transfer between the polymer matrix and the filler [15,16]. Several studies have shown that the addition of nanotubes causes an increase in hardness, stiffness, and strength of polyethylene matrix, which leads to an increase in the wear resistance of the material [5,6,14,15,17].

In the present study, bulk oriented UHMWPE films with a width of more than 2 mm and a drawing ratio (DR) of 35 were reinforced with multi-walled carbon nanotubes. The obtained bulk UHMWPE films had high tensile properties and excellent anti-friction properties. So, it is possible to recommend the use of the obtained films as a friction surface, for sliding bearings and/or polymer cups of the acetabular component, in order to reduce the amount of wear particles (debris) and increase the lifetime of the joint.

## 2. Materials and Methods

UHMWPE with a molecular weight of 1 × 10^6^ g/mol was purchased from “Kazanorgsynthesis Ltd.” (Kazan, Russia). Multi-walled carbon nanotubes (MWCNT) purchased from “Nanotechcenter Ltd.” (Tambov, Russia) were used as a filler. Their internal and external diameters were equal to 4–8 nm and 8–15 nm, respectively, and their length exceeded 2 mm. MWCNTs were treated by direct fluorination in order to increase the surface polarity and decrease agglomeration. MWCNT fluorination was carried out in a closed reaction vessel at a fluorine pressure of 0.7–0.8 bar at 150 °C over 2 h [18,19]. Fluorinated MWCNTs (FMWCNTs) were mixed with UHMWPE in a high-energy planetary ball mill APF-3 in 900-mL steel vials for 45 min. The diameter of the balls was 7–9.5 mm and the rotation speed was equal to 450 rpm. The concentration of FMWCNTs in UHMWPE was equal to 0.05, 0.1, 0.5, and 1 wt %.

The obtained UHMWPE/FMWCNT composite powders were transferred to the gel state before extrusion. P-xylene was used as a solvent (1.6 mL of solvent per 1 g of UHMWPE). The gel was extruded by a ram extruder UE-MSL (Extrusion Machinery Sales Ltd). A detailed description of the UHMWPE extrusion technique was previously described in Reference [20]. Then, the extruded gel was dried (xerogel) and rolled at 100 °C to reach a DR value of 2.5 by a calendar machine. Multi-stages of hot orientation of the UHMWPE films were carried out stepwise at temperatures of 110 °C (DR = 9), 120 °C (DR = 12), 130 °C (DR = 18), 140 °C (DR = 26), and 140 °C (DR = 35). The modes of thermo-orientation of UHMWPE films were previously developed as in Reference [20].

The structure of the UHMWPE xerogel (quasi-brittle fracture) and films after tensile strength measurement were examined by using a scanning electron microscope (SEM) VEGA 3 TESCAN (TESCAN, Brno, Czech Republic). Fracture surface of the UHMWPE xerogel was obtained by quasi-brittle fracture when the sample was stored in a liquid nitrogen (*T* = 77 K) for an hour and then broken. The polymer surface was coated with a Pt layer of 10–20 nm in thickness to avoid a charge accumulation magnetron deposition equipment JFC-1600 (JEOL, Tokyo, Japan) was used.

Thermal analysis of the samples was carried out by using a differential scanning calorimeter (DSC) NETZSCH DSC 204 F1 (NETZSCH, Selb, Germany) in argon atmosphere according to the ASTM D 3417–83. The key parameters for DSC curves processing were *T*_m_^onset^—the onset of the melting peak, *T*_m_—the melting temperature peak, and *T*_m_^end^—the end of the melting peak. The relative degree of crystallinity was calculated as the ratio of the experimental sample melting enthalpy compared to the completely crystallized polyethylene melting enthalpy, which is equal to 293 J/g [21].

Tensile strength of the bulk oriented films with different DR was measured by a Zwick/Roell Z020 (Zwick, Ulm, Germany) universal testing machine at a 10 mm/min loading rate. At least five measurements were used to obtain the tensile strength value for each film with a predetermined DR.

Vickers hardness tests of the films were performed by using the hardness tester MicroMet 5114 (BUEHLER, Lake Bluff, IL, USA). The hardness tests were carried out under 1 N indenter load and dwell time of 15 s, and 10 indentation points were chosen randomly.

A tribological study of the films was carried out by a cylinder-disk method at a sliding speed of 2.6 m/s in the mode of dry friction. The counter body was made of steel with a surface roughness no worse than *Ra* = 0.4. The counter body diameter was 100 mm. The load was varied over the 9.64 ÷ 32 N range. The specific wear rate was determined for a sliding distance of 2.355 km and a load of 32 N. The specific wear rate was calculated according to the Equation (1):

(1) $$I=\frac{V}{NL}$$

where *V*—the wear volume loss, *L*—the sliding distance, *N*—the normal load.

## 3. Results and Discussion

**Figure 1:**
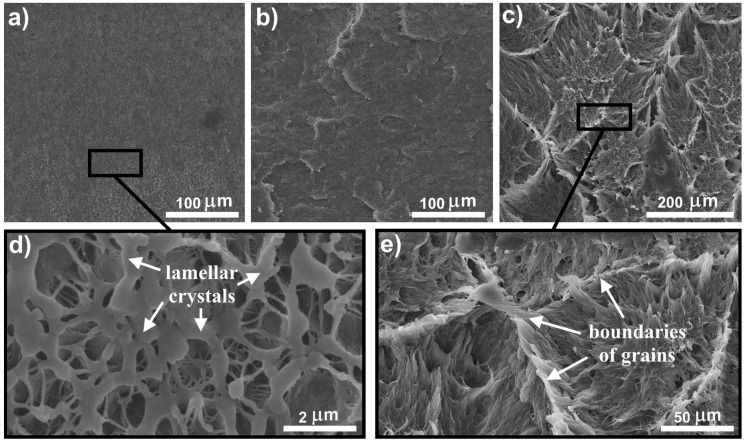
SEM of supramolecular structure of xerogel UHMWPE/FMWCNT with different contents of nanotubes (**a**) 0, 0.05, and 0.1 wt %, (**b**) 0.5 wt %, and (**c**) 1 wt %. The (**d**) scaled area shows the lamellar structure (content of FWMCNT 0, 0.05, and 0.1 wt %). The (**e**) scaled area shows the fibrillar structure and the boundaries grains of initial UHMWPE particles (content of FWMCNT 1 wt %). UHMWPE/FMWCNT, ultrahigh molecular weight polyethylene/fluorinated multi-walled carbon nanotubes.

The final properties of the UHMWPE oriented materials mainly depend on the supramolecular structure of the prepared xerogels and their drawability. Figure 1 shows the SEM micrograph of xerogels’ surfaces based on UHWMPE and UHMWPE/FMWCNT with different nanotube contents. The surfaces of xerogels, shown in Figure 1a, with a content of nanotubes of 0, 0.05, and 0.1 wt % have a homogeneous porous structure formed by the lamellar UHMWPE crystals, shown in Figure 1d. The lamellar crystals are joined together by fibrils. The space between the lamellar crystals is filled with macrovoids with a diameter of 0.5–1.5 μm. The macrovoids of xerogels have a positive effect on the processes of hot orientation of UHMWPE films, since it provides a free volume for rearranging the macromolecules of the polymer. The formed structure of xerogels UHMWPE with a FMWCNTs content up to 0.1 wt % provides a spinnability and shows the optimal way to obtain highly oriented films.

The structure of xerogels UHMWPE/FMWCNT with a nanotube content of 0.5 and 1 wt % is characterized by the preservation of the boundaries between the UHMWPE particles, see Figure 1b,c. In particular, the boundaries between UHMWPE particles are visible in xerogel UHMWPE/1% FMWCNT, Figure 1e. Apparently, at a concentration of FMWCNTs of more than 0.5 wt %, a critical amount of nanotubes accumulates at the boundaries of UHMWPE particles, which inhibits the diffusion of polyethylene macromolecules. This leads to the appearance of a defective structure at the interfaces of the UHMWPE particles (fusion defects), as a result of their poor interaction at the molecular level. Nanotubes accumulated on the surface of UHMWPE particles prevent the penetration of the solvent into the inside of the particle, and non-regular macrovoids in xerogels with a FMWCNTs content of 0.5 and 1 wt % form as a result. The accumulation of FMWCNTs on the surface of UHMWPE particles is associated with the method of their introduction into the UHMWPE matrix. Mixing in a high-energy planetary ball mill can lead to the distribution of filler particles but only over the UHMWPE particles’ surfaces [22,23].

**Figure 2:**
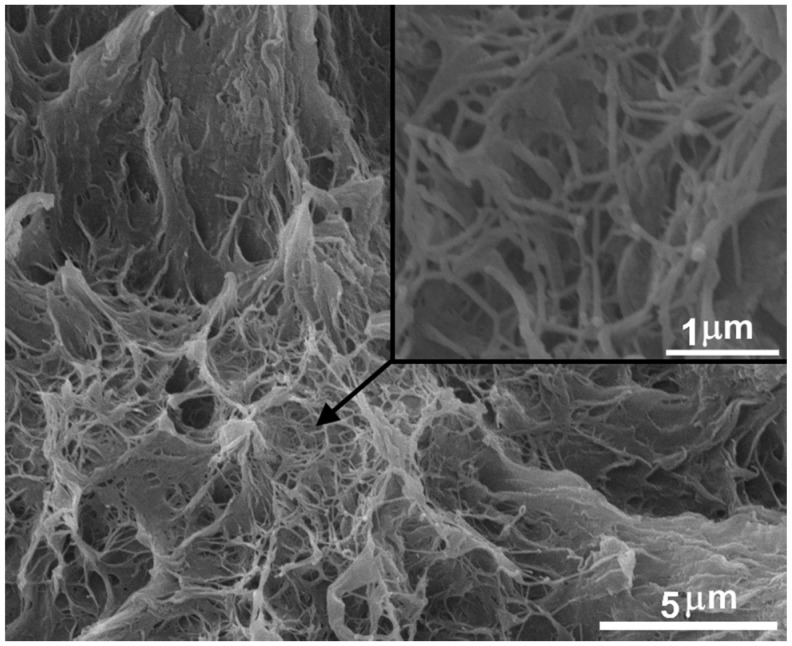
SEM of fibrillar structure of xerogel UHMWPE/1% FMWCNT.

The high concentration of FMWCNTs on the surface of polymer particles prevents the formation of a spinnable structure in UHMWPE and stimulates the formations of high amounts of fibrils, as shown in Figure 2. It is highly considered that the fibrillar structure in xerogel is caused by the lower amount of taut tie molecules revealed in the films. This prevents homogeneous stress distribution in the hot orientation process [24].

Table 1 shows the DSC data for xerogels and films based on UHMWPE and UHMWPE/FMWCNT with a DR of 35. The addition of FMWCNTs leads to an increase in the melting point and the degree of crystallinity of the xerogels. FMWCNTs can act as crystallization centers [25], and this leads to an increase in the degree of crystallinity. The increase in the degree of crystallinity may be accompanied by the increase in the dimensions of the crystalline phase, which leads to the increase in the melting temperature of the polymer. The role of FMWCNTs as centers of crystallization confirms the fact that the increase in the concentration of MWСNTs up to 0.5 wt % is accompanied by a proportional increase in the melting point and the degree of crystallinity. A further increase in the concentration of nanotubes in the xerogel does not lead to an increase in the melting point and the degree of crystallinity, due to the excessive accumulation of FMWCNTs on the surface of the polymer particles. Because the excessive concentration of FMWCNTs hinders interactions with UHMWPE macromolecules, some nanotubes no longer act as centers of crystallization.

**Figure 3:**
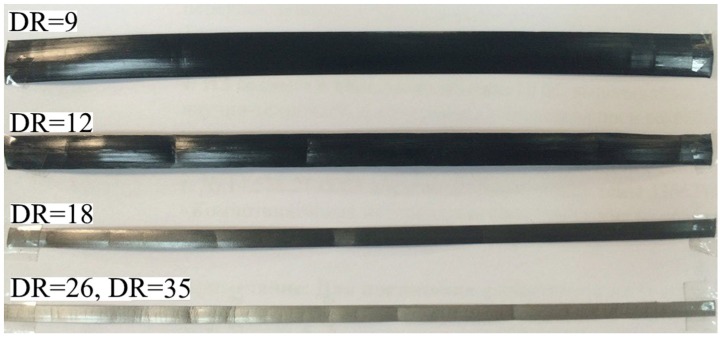
Photo of bulk oriented films based on UHMWPE/1% FMWCNT with different draw ratios (DR). DR is indicated in the figure.

All obtained films with a DR of 35 have a very high degree of crystallinity up to 95%. Such a high degree of crystallinity can be explained due to the orientation of macromolecules and their recrystallization (transformation of lamellar structure into fibrillar) in the process of hot drawing. The dependence of the crystallinity degree of films on the concentration of FMWCNTs is not observed. The high degree of crystallinity of films with a DR of 35 is accompanied by a change of color from black to gray, as shown in Figure 3. A similar change in color as well as the increase of the degrees of DR and crystallinity were observed in our earlier work [10], when the films became non-transparent and acquired a white color.

The results of the tensile tests are given in a comparison with films reinforced with initial MWCNTs (without fluorination), see Table 2. The non-filled UHMWPE films have a tensile strength of 0.9 GPa and an elastic modulus of 35 GPa. The introduction of FMWCNTs at a concentration of 0.05 wt % leads to an increase in the tensile strength by 10%. The elastic modulus remains practically unchanged. Nevertheless, the introduction of 0.05 wt % initial MWCNTs does not lead to a change in the mechanical properties. The increased concentration of initial and fluorinated MWCNTs in UHMWPE films leads to a decrease in the tensile strength and elastic modulus, see Table 2. Moreover, the addition of initial MWCNTs stimulates the reduction of mechanical properties dramatically. The elongation at the break for all the bulk oriented films was 4 ÷ 7%.

The reduction of the mechanical properties of films when introducing the initial MWCNTs can be explained by the appearance of poor interfacial bonding between the filler and the polymer. The absence of interfacial bonding and the accumulation of filler on the surface of UHMWPE particles are a result of MWСNTs performing as defects. With an increase in the concentration of initial MWCNTs, the number of defects increases, which is reflected in the proportional decrease in mechanical properties. We observed this in the bulk oriented UHMWPE films filled with initial nanotubes.

The fluorination of MWCNTs stimulates the formation of a strong interfacial interaction with macromolecules. As shown by the DSC data, the fluorination of MWCNTs increases the degree of crystallinity of xerogels, which is indicated by the formation of at least a good physical interfacial interaction between the polymer matrix and the filler. A good interfacial enhances the load transfer efficiency from the matrix to FMWCNTs, which leads to an increase in the mechanical properties.

The concentration of fluorinated nanotubes of 0.05 wt % in UHMWPE films is optimal for the achieved level of mixing. Nanotubes interact with macromolecules, which is reflected in the increase of mechanical properties. The increase in the concentration of FMWCNTs on the surface of UHMWPE particles leads to the accumulation of nanotubes that do not interact well with the UHMWPE macromolecules. In this case, some of the nanotubes begin to play the role of defects, as it was observed in the case of the initial nanotubes. The accumulation of the nanotubes on the surface of UHMWPE particles leads to the formation of a barrier, which prevents the self-diffusion processes between the UHMWPE macromolecules. Thus, defects are formed in the structure of the films at the boundaries of the initial UHMWPE particles, see Figure 1e, which reduces the mechanical properties of the films.

The highest decrease in the elastic modulus up to 24 GPa was demonstrated for the bulk oriented films filled with 1 wt % FMWCNTs. Such a high decrease in the elastic modulus could be attributed to the decrease in the number of taut tie molecules. As shown by the SEM method, see Figure 2, the introduction of 1 wt % FMWCNTs leads to the appearance of a fibrillar structure in the xerogel. The fibrillar structure formation in xerogel is probably caused by the lower amount of taut tie molecules revealed in an oriented film [24]. Taut tie molecules are very important constituents in highly oriented material structures. Taut tie molecules take part in the load redistribution between the crystalline blocks and influence the tensile strength and elastic modulus of highly oriented materials significantly [26,27].

The hardness of bulk oriented UHMWPE films increases by 27% after the formation of an oriented structure, see Table 2. As shown by the DSC data, the hot drawing of UHMWPE films was accompanied by a significant increase in the degree of crystallinity, which leads to an increase in the hardness. However, more effects of UHMWPE films related to the hardness occur with an increase in the FMWCNTs concentration. Upon the addition of FMWCNTs 1 wt %, there is an almost two-fold increase in the hardness of the films up to 10.3 HV.

**Figure 4:**
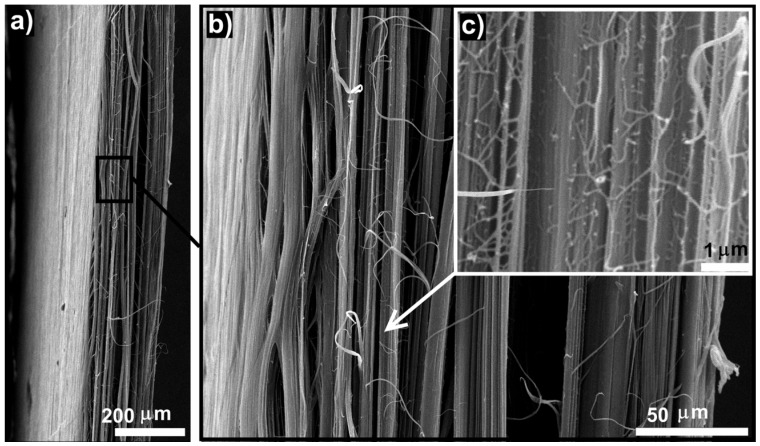
Typical fibrillar structure in the obtained bulk oriented films with different magnification. (**a**) 200 µm; (**b**) 50 µm; (**c**) 1 µm. The film’s axis is in the vertical direction.

Figure 4a,b show the SEM micrographs of the obtained bulk oriented UHMWPE/FMWCNT films with a well-defined fibrillar structure. Macrofibrils are connected by numerous transverse stressed microfibrils, Figure 4c, which are interfibrillar bonds. These microfibrils provide a connection between the structural elements in the bulk film. Transverse microfibrils do not lead to a film delamination into macrofibrils under the load.

The dependence of the dry friction coefficient of the bulk oriented films as a function of the FMWCNTs concentration and the load in the friction contact are shown in Table 3. The bulk oriented UHMWPE films without FMWCNTs exhibit an extremely low coefficient of friction in the range of 0.082 ÷ 0.124, depending on the applied load. The addition of FMWCNTs in the load range from 9.64 to 22.4 N has a feature of increasing the coefficient of friction. In the load range from 28.7 to 32 N, the coefficient of friction tends to be decreased. The behavior of the coefficient of dry friction for all the bulk oriented films is characterized by a general trend—an increase in the coefficient of dry friction with an increase in the load up to 19.2 N and a further decrease in the coefficient of dry friction with an increase in load. It is necessary to note that if films work in the zone of elastic deformation (load range from 9.64 to 22.4 N), nanotubes contribute to the increase of the coefficient of friction (COF). If films work in the zone of plastic deformation (load range from 28.7 to 32 N), nanotubes contribute to the reduction of the COF.

The COF has a dual molecular-mechanical nature, based on the adhesive and deformation components [28]. The dominance of the adhesive or the deformation of the components depends on the friction conditions and the material properties; therefore, there are cases of an increase [29,30] and decrease [31,32,33,34] in the COF with a rise in the contact load. In our work, the increase in the contact load up to 19.2 N was accompanied by an elastic deformation of the films that caused an increase in the COF due to the dominance of the deformed component. A further increase in the contact load had led to the plastic deformation of the surface. Then it was possible to reduce the COF by decreasing the dominant adhesion component (tangential stresses destroy molecular bonds whose growth is proportional to the increase in normal load).

**Figure 5:**
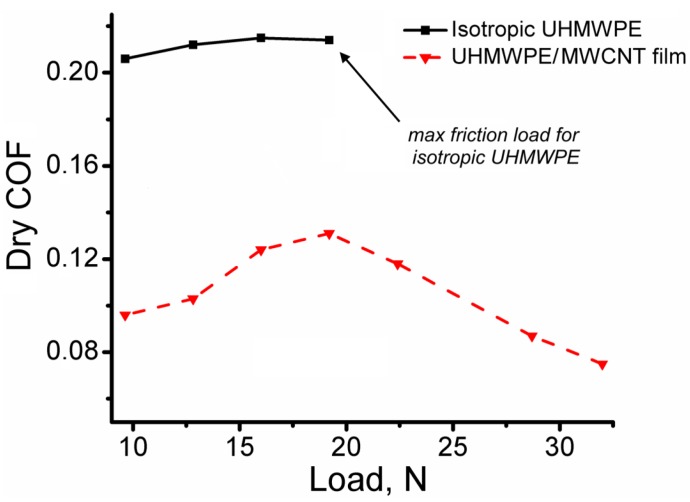
Dependence of the coefficient of dry friction on the load for traditional (conventional) UHMWPE with isotropic structure and bulk oriented UHMWPE/1% FMWCNT film. The traditional UHMWPE was prepared by the hot pressing method.

Figure 5 shows a plot of dry COF versus the load for traditional UHMWPE with isotropic structure and the bulk oriented UHMWPE/1% FMWCNT films. The bulk oriented UHMWPE/1% FMWCNT films have a dry COF that is more than two times lower compared to the traditional UHMWPE prepared by the hot pressing method. It is important to note that, under the selected conditions of tribological testing, the traditional UHMWPE with isotropic structure can operate up to a load of 19.2 N or a contact pressure of 1.7 MPa (contact pressure was calculated from the area of the wear spot at the end of the test). A friction load of more 19.2 N for traditional UHMWPE occurs in an intensive wear of the material. The bulk oriented UHMWPE/1% FMWCNT films can operate with a friction load of 32 N or contact pressure of 31.4 MPa, demonstrating an extremely low dry COF equal to 0.075.

**Figure 6:**
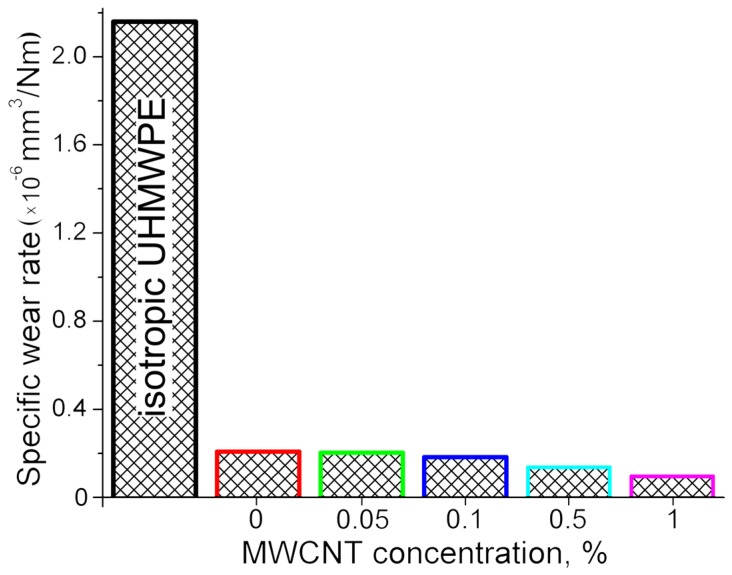
Specific wear rate for the isotropic UHMWPE and the oriented UHMWPE films with various concentrations of FMWCNTs.

The specific wear rate of the bulk oriented films, depending on the FMWCNTs concentration, is presented in Figure 6. For comparison, a specific wear rate for the isotropic UHMWPE measured at a load of 19.2 N was added to Figure 6, which clarifies that the specific wear rate decreases by one order 2.16 × 10^−6^ mm^3^/Nm for isotropic and 0.213 × 10^−6^ mm^3^/Nm for bulk oriented non-filled bulk films, after the formation of the oriented structure of UHMWPE. The addition of FMWCNTs and the increase of their concentrations help to improve the wear resistance of bulk oriented films. A maximum wear resistance is observed for the bulk oriented UHMWPE/1% FMWCNT films. An addition of FMWCNTs 1 wt % contributes to a two-fold reduction in the specific wear rate up to 0.098 × 10^−6^ mm^3^/Nm compared to the bulk oriented films without FMWCNTs.

The enormous increase in the wear resistance of the bulk oriented films compared to the traditional UHMWPE with isotropic structure is due to several reasons: (1) an increase in the mechanical properties; (2) the addition of FMWCNTs as antifriction filler; (3) the orientation of UHMWPE macromolecules in the direction of the sliding friction.

The mechanical properties of the material, especially the increase in the hardness of the friction surface, have a direct effect on the coefficient of friction and wear resistance of the material. The increase in the hardness and rigidity lead to a reduction in the volume of deformation of the friction surface of the films, thereby reducing the introduction of more rigid elements of the counter body surface into the softer surface of the polymer. This is directly reflected in the reduction in the dry COF of the films. The increase in the hardness and rigidity of the material allows the bulk oriented UHMWPE films to work at higher contact pressures while maintaining good antifriction properties. The increase in the strength of the films increases the fatigue strength of the surface elements, which in turn reduces the fatigue wear. The bulk oriented UHMWPE films have mechanical properties more than 10 times superior to that of isotropic UHMWPE. This sharp increase in the mechanical properties leads to a similar improvement in tribological properties.

Nanotubes have a high resistance to abrasion; therefore, those nanotubes which are naked the surface of the composite can help to reduce the abrasion. The addition of FMWCNTs leads to the increase of the hardness of bulk oriented films by up to twice as much, which in turn leads to a decrease in the deformation of components for the coefficient of friction.

The friction in the polyethylene matrix causes a molecular motion. In the case of sliding friction perpendicular to the alignment of macromolecules, the polymer chains have a tendency to orient in the direction of sliding, and demonstrate a high molecular motion. If there the sliding friction and the alignment of macromolecules coincide, the molecular motion will be lower. Therefore, PE chains have more shear deflection during perpendicular sliding than during parallel sliding, which corresponds to a higher shear strain energy [35], and that leads to an increase in the coefficient of friction. The high crystallinity of the polymer leads to the reduction of the possibility for molecular motion. Therefore, in the case of the parallel sliding friction, the formation of a high crystallinity and high orientation fibrillar structure of UHMWPE films leads to a significant decrease in the coefficient of friction. The fibrillar structure of the bulk oriented films has an increased fatigue resistance due to the high strength in this direction.

## 4. Conclusions

In this article, we obtained bulk oriented films based on UHMWPE and FMWCNTs with a DR of 35. It was shown that the introduction of FMWCNTs in a concentration of up to 0.1 wt % with the use of high energy ball milling promotes the production of xerogel with a fiber-form structure (spinnability), which is optimal for the production of films.

Initial MWCNTs (without fluorination) have a poor interfacial bonding with macromolecules of the polymer, and their introduction is accompanied by a decrease in tensile strength. The introduction of FMWCNTs at a concentration of 0.05 wt % in the bulk oriented UHMWPE films leads to an increase in the tensile strength by 10% as a result of the formation of a strong interfacial interaction. The strong interaction between the polymer and the filler was realized through the functionalization of carbon nanotubes by direct fluorination. However, an increase in the FMWCNTs concentration results in a decrease in tensile strength as a result of their excessive accumulation at the boundaries of UHMWPE particles.

Bulk oriented UHMWPE/1% FMWCNT films demonstrated an extremely low dry COF (0.075) with a contact load of 31 MPa (1.7 MPa for isotropic UHMWPE). The bulk oriented UHMWPE/FMWCNT films have a specific wear rate more than 10 times and less than the isotropic UHMWPE. The great increase of tribological properties is associated with: (1) a multiple increase in the mechanical properties of films, which makes them more rigid; (2) the formation of the fibrillar oriented structure; and (3) the presence of antifriction particles—carbon nanotubes. The obtained bulk oriented films can be used as a friction surfaces, for example, for metal-polymeric sliding bearings or polymer cups of the acetabular component.

## Figures and Tables

**Table 1:** DSC data of xerogels and films based on UHMWPE and UHMWPE/FMWCNT with a DR of 35.

Materials	*T_m_^onset^*, °C		*T_m_*, °C		*T_m_^end^*, °C		Crystallinity, %	
	Xerogel	Film	Xerogel	Film	Xerogel	Film	Xerogel	Film
UHMWPE reactor powders	121.9		141.6		144.5		75 ± 2	
UHMWPE without FMWCNT	116.9	138.4	133.6	143.5	140.4	150.3	69 ± 2	94 ± 2
UHMWPE/0.05% FMWCNT	123.1	137.9	139.8	143.4	143.8	149.6	73 ± 2	93 ± 2
UHMWPE/0.1% FMWCNT	124.7	137.7	140.9	143.2	147.2	148.6	75 ± 2	93 ± 2
UHMWPE/0.5% FMWCNT	126.5	137.7	141.1	143.5	148.7	152.2	78 ± 2	95 ± 2
UHMWPE/1% FMWCNT	126.6	139.3	141.7	143.9	148.8	153.8	77 ± 2	95 ± 2

**Table 2:** Mechanical properties of films based on UHMWPE and initial/fluorinated MWCNTs with a DR of 35.

Materials	Tensile Strength, MPa		Young’s Modulus, GPa		Elongation at Break, %		Hardness, HV
	Initial MWCNT	Fluorinated MWCNT	Initial MWCNT	Fluorinated MWCNT	Initial MWCNT	Fluorinated MWCNT	
Isotropic UHMWPE	27 ± 2		0.72 ± 0.02		>300		4.3 ± 0.1
UHMWPE without MWCNT	920 ± 13		35 ± 0.365		4.5 ± 0.3		5.5 ± 0.1
UHMWPE/0.05% MWCNT	913 ± 21	1020 ± 23	35 ± 0.24	36 ± 0.27	4.3 ± 0.3	5.85 ± 0.3	5.8 ± 0.1
UHMWPE/0.1% MWCNT	721 ± 56	960 ± 41.1	31 ± 0.456	36 ± 0.47	4.1 ± 0.5	5.75 ± 0.4	7.1 ± 0.2
UHMWPE/0.5% MWCNT	635 ± 46	835 ± 31.8	31 ± 0.75	35 ± 0.52	4.5 ± 0.5	7.5 ± 0.4	8.4 ± 0.2
UHMWPE/1% MWCNT	604 ± 41	695 ± 27.3	31 ± 0.23	24 ± 0.31	4.1 ± 0.3	5 ± 1.1	10.3 ± 0.2

**Table 3:** Dry COF of bulk oriented films depending on the concentration of the FMWCNTs and the load in the contact of friction

Load, N						
9.64	12.82	16.0	19.2	22.4	28.7	32.0
**UHMWPE without FMWCNT**						
0.082 ± 0.001	0.103 ± 0.002	0.124 ± 0.003	0.124 ± 0.001	0.118 ± 0.002	0.106 ± 0.001	0.103 ± 0.004
**UHMWPE/0.05% FMWCNT**						
0.109 ± 0.002	0.113 ± 0.001	0.124 ± 0.004	0.117 ± 0.001	0.112 ± 0.003	0.092 ± 0.002	0.095 ± 0.002
**UHMWPE/0.1% FMWCNT**						
0.109 ± 0.001	0.123 ± 0.002	0.132 ± 0.004	0.130 ± 0.002	0.124 ± 0.002	0.092 ± 0.001	0.095 ± 0.003
**UHMWPE/0.5% FMWCNT**						
0.07 ± 0.002	0.093 ± 0.001	0.116 ± 0.002	0.123 ± 0.003	0.118 ± 0.001	0.101 ± 0.003	0.103 ± 0.002
**UHMWPE/1% FMWCNT**						
0.096 ± 0.001	0.103 ± 0.002	0.124 ± 0.001	0.131 ± 0.001	0.118 ± 0.002	0.087 ± 0.002	0.075 ± 0.002
